# 胸腺原发神经内分泌癌所致库欣综合征手术治疗及预后分析

**DOI:** 10.3779/j.issn.1009-3419.2015.07.12

**Published:** 2015-07-20

**Authors:** 力 李, 野野 陈, 单青 李, 洪生 刘, 诚 黄, 应之 秦

**Affiliations:** 100730 北京，中国医学科学院北京协和医院胸外科 Department of Thoracic Surgery, Endocrine Key Laboratory of Ministry of Health, Peking Union Medical College Hospital, Chinese Academy of Medical Science, Beijing 100730, China

**Keywords:** 神经内分泌癌, 胸腺, 库欣综合征, 手术治疗, 预后分析, Neuroendocrine carcinoma, Thymus, Cushing syndrome, Surgical procedures, Prognosis

## Abstract

**背景与目的:**

胸腺原发神经内分泌癌为一种罕见疾病，部分肿瘤可以分泌促肾上腺皮质激素导致库欣综合征，手术切除肿瘤是治疗的关键。本研究回顾性总结并探讨胸腺原发神经内分泌癌所致库欣综合征临床诊断及手术治疗方法，并行预后分析，提高对此罕见病种的认识及诊疗水平。

**方法:**

回顾性分析北京协和医院1987年11月-2013年6月间收治的14例由胸腺原发神经内分泌癌导致的库欣综合征患者的临床资料。

**结果:**

14例患者，男性8例，女性6例，中位年龄29岁，中位病程4.0个月(1个月-44个月)，术前多项内分泌检查及影像学检查诊断并定位胸腺病变，经手术切除后血皮质醇及血促肾上腺皮质激素均下降(*P* < 0.01)。围手术期死亡1例。经术后综合治疗及随访，中位生存期为38个月。

**结论:**

胸腺原发神经内分泌癌所致库欣综合征为一种罕见疾病，侵袭性强，目前治疗效果欠佳。早期诊断及早期治疗对临床医师而言存在很大难度，胸部增强计算机断层扫描(computed tomography, CT)是诊断并定位的重要手段，需要多种检查手段及多学科医师共同会诊并制定以手术为主的综合诊治方案。

## Abstract (en)

**Background and objective:**

Primary neuroendocrine carcinoma of thymus (pNECT) is a rare thymic neoplasm. Some pNECTs could produce an adrenocorticotropic hormone and cause Cushing syndrome (CS). The aim os this study is to discuss the diagnostic technique and surgical management of pNECT-caused CS and analyze prognosis factors to improve the clinical experience of the disease.

**Methods:**

The outcome of surgery and follow-up of 14 cases (eight males and six females) of pNECT-caused CS were retrospectively analyzed from November 1987 to June 2013.

**Result:**

The median age of the patients was 29, and the median duration of the disease was four months (1 month-44 months). All cases exhibited clinical evidence for the diagnosis of CS, and thoracic computed tomography (CT) was used to detect thymic tumors. Surgical treatment significantly decreased the concentration of both serum cortisol and adrenocorticotropic hormone (*P* < 0.01) but caused one death in the perioperative period. With multidisciplinary therapy, the median survival was 38 months.

**Conclusion:**

pNECT-caused CS is a rare disease with aggressive characteristics and unclear prognosis. Early diagnosis and therapy is a challenge for clinicians. Thoracic CT is important for disease location and preoperative evaluation and should be routinely applied to all CS patients to allow early surgery and improved prognosis.

胸腺原发神经内分泌癌(primary neuroendocrine carcinoma of thymus, pNECT)为一种罕见的恶性病变，约占胸腺上皮肿瘤2%-5%^[1-5]^。自1972年Rosai等^[6]^首次报道至今，文献报道总病例数仅有数百例。约10%-30%的pNECT可以分泌促肾上腺皮质激素(adrenocorticotropin hormone, ACTH)，从而导致库欣综合征(Cushing syndrome, CS)，这种由非垂体肿瘤异位分泌ACTH导致CS也称为异位ACTH综合征(ectopic adrenocorticotropic hormone syndrome, EAS)。

pNECT导致CS为极为罕见的疾病，目前对其诊治经验仅仅来自于病案报告及小规模的病例总结，缺乏诊治指南。本研究回顾性分析北京协和医院经手术治疗并由病理证实的pNECT导致CS病例，探讨这一罕见疾病的临床特点及外科治疗经验，分析潜在的预后因素。

## 1 资料与方法

回顾性分析1987年11月-2013年6月间在北京协和医院诊治确定为pNECTs导致CS并行手术治疗的患者资料。全组收集临床表现、实验室检查，影像学检查以及手术治疗、病理结果、辅助治疗及随访情况的相关资料，并对治疗情况及预后进行分析。应用SPSS 19.0统计分析软件。计量资料数据符合正态分布时，以均数±标准差表示；数据为非正态分布时，以中位数表示。采用*Kaplan-Meier*法分析生存期，生存期计算自手术日期开始到死亡日期或末次随访日期。采用*Log-rank*单因素分析对生存期的影响因素。以*P* < 0.05为差异有统计学意义。

## 2 结果

### 2.1 一般资料

1987年11月-2013年6月间经北京协和医院手术后病理证实为pNECT共51例，其中导致CS共14例，占27.4%。男性8例，女性6例，男女比为1.33:1。本组14例患者首次就诊时的中位年龄为29.0岁(13岁-46岁)(表 1)。

**1:** 手术患者术前临床特点 Clinical characteristics of surgical patients before operation

Characteristics	Value
Median age (yr)	29.0 (13-46)
Male/Female	1.33:1 (8:6)
Median duration (mo)	4 (1-44)
Symptom and sign	
Hypertesion	8/14 (57.1%)
Facial plethora	10/14 (71.4%)
Central obesity	9/14 (64.3%)
Moon face	10/14 (71.4%)
Buffalo bump	10/14 (71.4%)
Proximal muscle weakness	8/14 (57.1%)
Biological data	
Hyperglycemia	9/14 (64.3%)
Hypokalemia	13/14 (92.8%)
Serum cortisol elevation	14/14 (100.0%)
UFC elevation	14/14 (100.0%)
Plasma ACTH elevation	14/14 (100.0%)
LDDST negative	14/14 (100.0%)
HDDST negative	13/14 (92.8%)
IPSS no centroperipheral gradient	4/4 (100.0%)
Imaging	
Abnormal pituitary MRI or CT	3/14 (21.4%)
Abnormal adrenal gland CT scan	14/14 (100.0%)
Thoracic CT detection tymic mass	14/14 (100.0%)
^111^In-octreotide scintigraphy	6/7 (86%)
Unnecessary surgical intervention	
Adrenalectomy	2/14 (14.3%)
Hypophysectomy	2/14 (14.3%)
Saddle area radiotherapy	1/14 (7.1%)
UFC: urinary free cortisol; ACTH: adrenocorticotropic hormone; LDDST: low doses of dexamethasone test; HDDST: high doses of dexamethasone test; IPSS: inferior petrosal sinus sampling; CT: computed tomography; MRI: magnetic resonance image.	

### 2.2 症状、体征及实验室检查

14例患者从起病到确诊CS中位时间为4.0个月(1个月-44个月)，其中10例同时确诊EAS并发现行胸腺病变，而4例诊断CS时未考虑EAS诊断，延迟诊断EAS时间分别为5个月、30个月、33个月、44个月。全组患者均存在皮质醇增多症的表现：临床上主要有高血压、糖尿病、多血质面容、向心性肥胖、满月脸和水牛背等症状和体征(表 1)。所有患者血皮质醇水平均升高且伴随生理节律消失，平均值(43.8±19.2)μg/dL(最低23.8 μg/dL，最高87.0 μg/dL)，所有患者的血ACTH水平均升高，中位值197.0pg/mL(最低79.3 pg/mL，最高1, 033.0 pg/mL)。小剂量地塞米松试验均不被抑制，而大剂量地塞米松试验有1例患者被抑制。4例患者行岩下静脉窦采血，提示与外周血的ACTH比值小于2，梯度消失，支持EAS的诊断。

### 2.3 影像学检查

所有患者均由计算机断层扫描(computed tomography, CT)发现胸腺占位病变。本组中有7例患者行奥曲肽显像检查，其中6例患者(86%)胸腺区病变显影，位置与CT检查相符。肾上腺CT提示所有患者均存在不同程度的肾上腺增生，鞍区影像学有3例患者怀疑有微腺瘤。

### 2.4 前期垂体及靶器官手术

2例患者诊断CS时怀疑EAS诊断，但胸片(当时未行CT检查)未发现异常，因病情较重为控制高皮质醇血症行双侧肾上腺切除，病理均为肾上腺皮质弥漫性增生。3例患者怀疑垂体病变，1例行鞍区放疗，另2例行鞍区探查术，术后病理1例垂体细胞增生，另1例为垂体微腺瘤。

### 2.5 胸腺肿瘤手术方式及病理结果

从表 2可见，本组患者均行胸腺肿瘤切除术，11例经正中开胸，3例经右后外侧切口开胸；2例因外侵严重行姑息性切除，其余为完整切除。10例(71.4%)病变出现外侵。4例因肿物侵犯无名静脉或上腔静脉行上腔静脉或无名静脉切除重建，3例患者行单侧膈神经切除。1例患者术后2周因严重感染死于重症监护病房。其余病例未出现围手术期死亡或并发症。肿瘤的最大径线中位数为3.8 cm(2.5 cm-10.5 cm)。Masaoka-Koga分期^[7]^为Ⅰ期1例，Ⅱ期4例，Ⅲ期2例，Ⅳ期7例。组织病理学检查，4例为典型类癌，10例为不典型类癌，本组患者中未发现分化差的如小细胞或大细胞神经内分泌癌。有8例标本行ACTH免疫组化染色，7例为阳性表现(图 1)。

**1:**
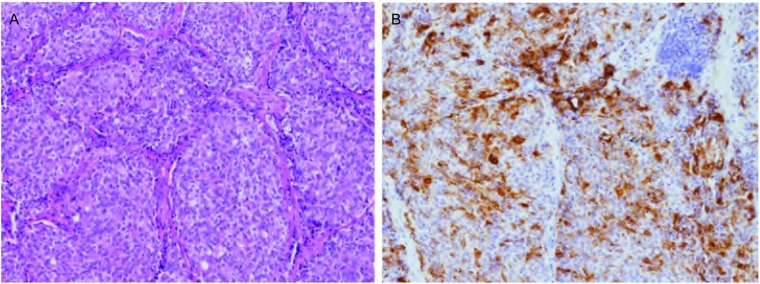
病理图片。A：苏木精-伊红染色，呈典型的巢状分布的神经内分泌细胞团(×150)；B：促肾上腺皮质激素免疫组化染色呈阳性(×150)。 Pathological image. A: Hematoxylin-eosin staining, typical nesting distribution consists of neuroendocrine cells (×150); B: Immunohistochemical staining: Positive for adrenocorticotropic hormone (×150).

**2:** 病理、分期及随访 Pathology, staging and follow-up

No.	Tumor size (cm)	LN	Aggresive	Vascular reconstruction	Masaoka staging	Pathology	Ad	Follow-up (mo)
1	3	7/8	-	Nd	Ⅳ	ATC	Nd	33
2	10	Nd	+	Repatching of SVC	Ⅳ	TC	Nd	10
3	3	7/8	+	Nd	Ⅳ	ATC	Ra	24
4	6	Nd	-	Nd	Ⅱ	TC	Ra	41
5	3	0/0	-	Nd	Ⅰ	TC	Nd	168
6	3	0/0	+	Nd	Ⅱ	ATC	Nd	66
7	3	0/0	+	Nd	Ⅱ	ATC	Ra	36
8	11	0/0	+	Double Ⅳ-RA bypass	Ⅲ	ATC	Nd	21
9	3	0/2	+	Nd	Ⅳ	ATC	Ra+C	15
10	4	8/8	-	Nd	Ⅳ	TC	Ra+C	38
11	4	0/0	+	Nd	Ⅱ	ATC	Ra	45
12	5	Nd	+	Nd	Ⅳ	ATC	Ra+C	96
13	7	17/17	+	Double Ⅳ-RA bypass	Ⅳ	ATC	Nd	0.5
14	5	0/0	+	Left Ⅳ-RA bypass	Ⅲ	ATC	Nd	26
SVC: superior vena cava; LN: lymph node; Ⅳ-RA bypass: innominate vein -right atrium bypass; TC: typical carcinoid; ATC: atipical carcinoid; Nd: not done; Ad: adjuvant therapy; Ra: radiotherapy; C: chemotherapy.								

### 2.6 术后治疗及随访与预后分析

术前术后血游离皮质醇平均值分别为(46.2±18.4)μg/dL及(11.3±7.0)μg/dL(配对*t*检验，*P* < 0.001，因术后1例患者死亡未测血皮质醇，有效配对数13例)，血ACTH中位值分别为197.0pg/mL及86.0pg/mL(非参数检验，*P*=0.002)。术后6例患者未进行辅助治疗，这其中除1例围手术期死亡外，1例于术后10个月死于肿瘤转移复发，另4例手术至末次随访时间分别为21个月、33个月、66个月及168个月，末次随访时无复发。8例患者行辅助治疗，其中6例为单纯纵隔区放疗，2例除放疗外还行化疗(依托泊苷+顺铂方案)，4例分别于术后第15、26、38、41个月死亡，两例于36个月、45个月后病情进展，建议进一步治疗，但患者未再随访，还有2例随访24个月、96个月无复发。采用*Kaplan-Meier*法计算生存期，本组患者中位生存期为38个月。应用*Log-rank*单因素分析对下列因素分析：性别(*P*=0.138)、年龄(≥中位年龄与 < 中位年龄比较)(*P*=0.730)、前期是否行垂体或靶腺手术(*P*=0.839)、肿瘤大小( > 5 cm与≤5 cm)(*P*=0.234)、病理类型(典型类癌与不典型类癌比较)(*P*=0.692)、肿瘤分期Ⅰ期+Ⅱ期及Ⅲ期+Ⅳ期(*P*=0.127)、术后是否行辅助治疗(*P*=0.279)，均未发现对生存期的影响有统计学差异。

## 3 讨论

胸腺神经内分泌癌为一种罕见的恶性肿瘤，2004年病理分型分为分化好的神经内分泌癌(包括类癌及不典型类癌)以及分化差的神经内分泌癌(包括小细胞及大细胞癌)。本组患者中无小细胞及大细胞癌，可能因为小细胞及大细胞癌本身就极为罕见，而且本回顾性研究入组患者为手术切除患者，但小细胞及大细胞因为恶性程度更高，发展快，发现时常为穿刺诊断后行辅助治疗。pNECT约有10%-30%可分泌ACTH从而导致CS。有多种非垂体的实体肿瘤，大多数为神经内分泌肿瘤，如支气管类癌、胸腺类癌、胰岛细胞癌、嗜铬细胞瘤等等，可以分泌ACTH，从而导致EAS^[8, 9]^。胸腔来源的肿瘤大约占到所有EAS病变来源的2/3左右，胸腺是除肺以外最主要的EAS肿瘤发生部位^[8-10]^。北京协和医院曾经报道过支气管肺类癌导致的库欣综合征^[11]^，与肺类癌相比，pNECT恶性程度更高，侵袭及转移的可能性更大，因此治疗上难度更大^[2, 7]^。本组患者占同期所有EAS患者的19.4%，与文献报告相近。由于其罕见性，目前国内外文献均为病例报告或小规模病例总结^[2, 12, 13]^，缺乏相应的诊治指南，诊断及治疗上常常困扰临床医师，漏诊、误诊病例较多。

诊断方面，同所有CS诊断一样，首先要通过化验确定高皮质血症，诊断CS，再区分垂体来源与非垂体来源。本组患者均通过化验确定为高皮质血症，并且小剂量地塞米松试验不被抑制，CS诊断明确。EAS患者大剂量地塞米松试验多数不被抑制，而本组有1例被抑制(7.1%)，明显低于文献报道的20%-40%^[8, 9]^，我院曾报道的肺类癌所导致的EAS大剂量地塞米松试验被抑制比例为4/13(30.8%)，这一特点是否是胸腺与肺来源EAS的区别还有待进一步研究。岩下静脉窦采血查ACTH梯度可作为定性诊断方法之一^[14]^，当中心与外周ACTH比值小于2，即我们所谓的梯度消失，则考虑为EAS，本组4例患者行此检查，比值均小于2。但由于这一操作为有创操作且对设备及操作者经验要求严格，因此很难广泛开展。

定性诊断考虑EAS后，定位则成为最终诊断并治疗的关键。作为发现胸腺肿瘤的方法，胸部CT无疑为最佳手段，胸片常常会因为心脏大血管以及脊柱等等的影响而无法辨清较小的纵隔肿物。但由于此类患者主诉症状多为内分泌相关症状，缺乏肿瘤局部症状，因此胸部CT常常不作为常规检查项目。本组中共有4例患者在诊断CS时未能诊断出EAS(28.6%)，2例是初诊时未考虑EAS未查胸部影像，而另2例是在怀疑EAS但胸片未见异常而漏诊的。这些患者在无法明确诊断的情况下进行了垂体或肾上腺的有创治疗，最终疗效不佳进一步检查胸部CT才最终发现胸腺病变，但分别延迟5个月、30个月、33个月、44个月。由于胸片上胸腺区较小病变容易被胸骨、脊柱、心脏大血管等遮挡，因此胸片常常漏诊(图 2)，如果在怀疑EAS同时查胸部CT，就能更及时发现胸腺肿瘤而达到早期诊断并治疗的目的，也能避免不必要的肾上腺或垂体的有创操作。另外，由于pNECT的外侵性很强，常常侵犯周围血管尤其是无名静脉及上腔静脉，胸部增强CT还可以判断肿瘤外侵的情况以及手术的可行性。因此我们建议，对于所有怀疑EAS的患者，甚至对于所有CS的患者，胸部增强CT应作为常规检查项目。

**2:**
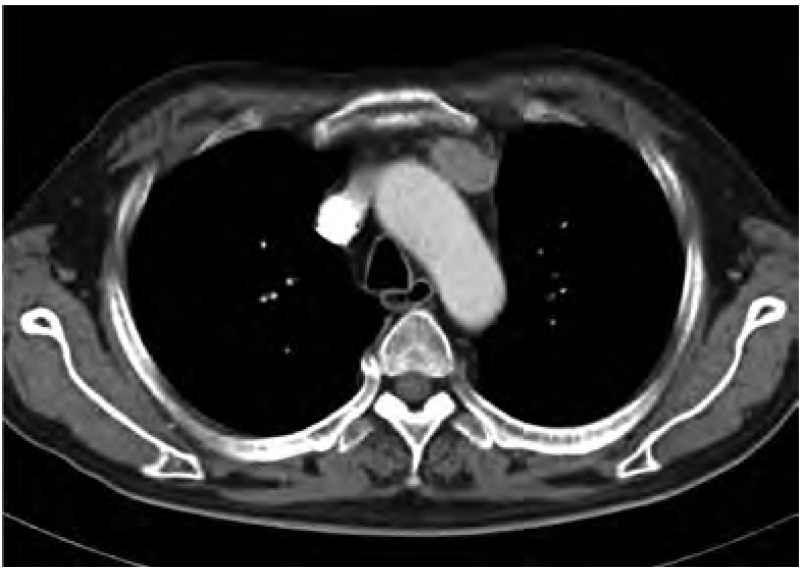
增强CT：可见胸腺类圆形病变，边界较光整，胸片上容易被主动脉弓及中轴骨的阴影遮挡。 Contrast-enhanced CT: Oval mass in thymic area with a clear boundary, which might be overlapping by opaque of aortic arch and axial bones in chest X-ray.

诊断明确后，首选治疗是手术切除肿瘤^[8]^，本组患者全经手术切除肿瘤，手术疗效明显，术前术后血游离皮质醇平均值分别为(46.2±18.4)μg/dL及(11.3±7.0)μg/dL(*P* < 0.001)，血ACTH中位值分别为197.0 pg/mL及86.0pg/mL(非参数检验，*P*=0.002)，说明手术切除异位ACTH来源肿瘤的重要性。对于pNECT而言，为降低局部复发率，首选的治疗方案是全胸腺切除^[15, 16]^。正中开胸不仅能切除全胸腺、周围脂肪以及周围受累的纵隔结构，且能行淋巴结清扫或采样行病理分期，因而是目前推荐的首选手术入路^[17]^。由于EAS治疗的复杂性，且由于内分泌因素对手术操作及术后恢复的影响，如感染风险增高、患者自身存在骨质疏松等，需根据患者的具体情况以及肿瘤生长部位、与周围血管脏器的关系等因素选择更利于操作的手术途径及手术方式。本组中，3例是右侧开胸，目前认为右侧开胸处理胸腺左侧上极存在一定困难，但对于肿瘤本身可达到完整切除，本组中随访时间最长且无复发的1例即为右侧开胸手术切除的患者。近来还有回顾性病例总结报道认为经右侧胸腔镜治疗早期尤其是Ⅰ期胸腺肿瘤，与正中开胸比较，有创伤小、住院时间短等优势，且复发率无差异等优势。因此，如能做到早期发现，对于pNECT所导致的EAS，胸腔镜手术治疗也可作为备选方法之一，对于这些长期内分泌激素异常的患者，可明显降低手术创伤，降低手术并发症的风险，但目前还缺乏远期随访数据的支持。另外，对于pNECT而言，辅助治疗的效果并不乐观，目前已有的研究认为，放疗可以降低局部复发率但不能改善远期存活率，而化疗方面，文献^[16, 18]^报道多数NECT都对目前的标准化疗方案都不敏感。因此早期发现并行手术切除仍是提高此类疾病治疗的关键点。

美国流行病监督及最终结果资料库(Surveillance, Epidemiology, and End Results, SEER)2010年总结的pNECT总体的中位生存期为64个月，发现时为局部进展期的患者中位生存期为59个月，而出现转移的患者中位生存期仅35个月^[19]^。本组患者的中位生存期为38个月，分析其原因，本组患者外侵率高达71.4%，而Masaoka-Koga分期Ⅲ期及Ⅳ期患者达64.3%(9/14)，本组患者中位生存期偏低可能与此相关。有学者提出合并CS的pNECT较无CS的pNECT预后差，可能因为具备异位分泌ACTH功能的pNECT侵袭性更强、转移发生率更高，也可能因为CS患者本身高皮质血症所导致的内分泌代谢异常所致，需要更多的病例总结及进一步研究证实。我们选取了性别、年龄、肿瘤大小、前期是否行垂体或靶腺手术、病理类型、肿瘤分期、术后是否进行辅助治疗等多个变量，进行*Log-rank*单因素分析，结果未能找到对生存期有影响的变量。仅仅在性别及肿瘤分期因素上*P*值接近0.1，似乎有一些倾向性，需要更多病例数及更长时间随访才能获得更准确的结论。

总之，pNECT异位分泌ACTH导致的CS为一种罕见的疾病，涉及多学科综合治疗，此类肿瘤侵袭性强，转移发生早，早期发现并明确诊断难度大，目前治疗效果尚不满意。对于所有CS患者，胸部CT应作为常规检查项目，更早发现异位分泌ACTH的病灶，以便早期手术切除肿瘤，改善预后。

## References

[1] Yao JC, Hassan M, Phan A (2008). One hundred years after "carcinoid": epidemiology of and prognostic factors for neuroendocrine tumors in 35, 825 cases in the United States. J Clin Oncol.

[2] Neary NM, Lopez-Chavez A, Abel BS (2012). Neuroendocrine ACTH-producing tumor of the thymus--experience with 12 patients over 25 years. J Clin Endocrinol Metab.

[3] Oberg K, Hellman P, Kwekkeboom D (2010). Neuroendocrine bronchial and thymic tumours: ESMO Clinical Practice Guidelines for diagnosis, treatment and follow-up. Ann Oncol.

[4] Strobel P, Zettl A, Shilo K (2014). Tumor genetics and survival of thymic neuroendocrine neoplasms: A multi-institutional clinicopathologic study. Genes Chromosomes Cancer.

[5] Crona J, Bjorklund P, Welin S (2013). Treatment, prognostic markers and survival in thymic neuroendocrine tumours. a study from a single tertiary referral centre. Lung Cancer.

[6] Rosai J, Higa E (1972). Mediastinal endocrine neoplasm, of probable thymic origin, related to carcinoid tumor. Clinicopathologic study of 8 cases. Cancer.

[7] Modlin IM, Lye KD, Kidd M (2003). A 5-decade analysis of 13, 715 carcinoid tumors. Cancer.

[8] Alexandraki KI, Grossman AB (2010). The ectopic ACTH syndrome. Rev Endocr Metab Disord.

[9] Isidori AM, Lenzi A (2007). Ectopic ACTH syndrome. Arq Bras Endocrinol Metabol.

[10] Isidori AM, Kaltsas GA, Pozza C (2006). The ectopic adrenocorticotropin syndrome: clinical features, diagnosis, management, and long-term follow-up. J Clin Endocrinol Metab.

[11] Zhu Y, Lu L, Li NS (2010). Ectopic ACTH syndrome due to bronchopulmonary carcinoid tumor. Ai Zheng Jin Zhan.

[12] Wang WQ, Zhao HY, Chen Y (2003). Ectopic ACTH caused by thymus carcinoid. Zhonghua Nei Fen Mi Dai Xie Za Zhi.

[13] Tan S, Zhang QG, Zhang L (2005). Sugrical treatment for ectopic ACTH syndrome caused by thymus carcinoid. Zhonghua Xiong Xin Xue Guan Wai Ke Za Zhi.

[14] Isidori AM, Kaltsas G, AMohammed S (2003). Discriminatory value of the low-dose dexamethasone suppression test in establishing the diagnosis and differential diagnosis of Cushing's syndrome. J Clin Endocrinol Metab.

[15] Cardillo G, Rea F, Lucchi M (2012). Primary neuroendocrine tumors of the thymus: a multicenter experience of 35 patients. Ann Thorac Surg.

[16] Ruffini E, Oliaro A, Novero D (2011). Neuroendocrine tumors of the thymus. Thorac Surg Clin.

[17] Rena O, Filosso PL, Maggi G (2003). Neuroendocrine tumors (carcinoid) of the thymic gland. Ann Thorac Surg.

[18] Cardillo G, Treggiari S, Paul MA (2010). Primary neuroendocrine tumours of the thymus: a clinicopathologic and prognostic study in 19 patients. Eur J Cardiothorac Surg.

[19] Gaur P, Leary C, Yao JC (2010). Thymic neuroendocrine tumors: a SEER database analysis of 160 patients. Ann Surg.

